# Exercise-induced Changes in Central Adiposity During an RCT: Effect of Exercise Dose and Associations With Compensation

**DOI:** 10.1210/clinem/dgad696

**Published:** 2023-11-29

**Authors:** James L Dorling, John W Apolzan, Neil M Johannsen, Diana M Thomas, Christoph Höchsmann, Daniel S Hsia, Corby K Martin

**Affiliations:** Human Nutrition, School of Medicine, Dentistry and Nursing, College of Medical, Veterinary and Life of Sciences, University of Glasgow, Glasgow G31 2ER, UK; Pennington Biomedical Research Center, Baton Rouge, LA 70808, USA; Pennington Biomedical Research Center, Baton Rouge, LA 70808, USA; School of Kinesiology, Louisiana State University, Baton Rouge, LA 70803, USA; Department of Mathematical Sciences, United States Military Academy, West Point, NY 10996, USA; TUM School of Medicine and Health, Technical University of Munich, Munich 80809, Germany; Pennington Biomedical Research Center, Baton Rouge, LA 70808, USA; Pennington Biomedical Research Center, Baton Rouge, LA 70808, USA

**Keywords:** physical activity, weight loss/reduction, abdominal obesity, visceral fat, energy intake, body composition

## Abstract

**Context:**

Exercise can decrease central adiposity, but the effect of exercise dose and the relationship between central adiposity and exercise-induced compensation is unclear.

**Objective:**

Test the effect of exercise dose on central adiposity change and the association between central adiposity and exercise-induced weight compensation.

**Methods:**

In this ancillary analysis of a 6-month randomized controlled trial, 170 participants with overweight or obesity (mean ± SD body mass index: 31.5 ± 4.7 kg/m^2^) were randomized to a control group or exercise groups that reflected exercise recommendations for health (8 kcal/kg/week [KKW]) or weight loss and weight maintenance (20 KKW). Waist circumference was measured, and dual-energy X-ray absorptiometry assessed central adiposity. Predicted weight change was estimated and weight compensation (weight change – predicted weight change) was calculated.

**Results:**

Between-group change in waist circumference (control: .0 cm [95% CI, −1.0 to 1.0], 8 KKW: −.7 cm [95% CI, −1.7 to .4], 20 KKW: −1.3 cm [95% CI, −2.4 to −.2]) and visceral adipose tissue (VAT; control: −.02 kg [95% CI, −.07 to .04], 8 KKW: −.01 kg [95% CI, −.07 to .04], 20 KKW: −.04 kg [95% CI, −.10 to .02]) was similar (*P* ≥ .23). Most exercisers (82.6%) compensated (weight loss less than expected). Exercisers who compensated exhibited a 2.5-cm (95% CI, .8 to 4.2) and .23-kg (95% CI, .14 to .31) increase in waist circumference and VAT, respectively, vs those who did not (*P* < .01). Desire to eat predicted VAT change during exercise (β = .21; *P* = .03).

**Conclusion:**

In the presence of significant weight compensation, exercise at doses recommended for health and weight loss and weight maintenance leads to negligible changes in central adiposity.

More than two-thirds of the US population lives with overweight or obesity, which are characterized by an elevated body mass index (BMI) (1). Overweight and obesity are key risk factors for cardiometabolic disease development (2), and an established link between BMI and cardiometabolic disease risk exists (3). However, central adiposity, primarily visceral adipose tissue (VAT), is strongly associated with metabolic disease risk factors (eg, high blood glucose (4) and dyslipidemia (5)), cardiometabolic conditions (eg, metabolic syndrome, type 2 diabetes (6), and cardiovascular disease (5)), and mortality (7). Additionally, some have shown central adiposity is more strongly related to metabolic diseases such as type 2 diabetes than fat stored in other regions (3, 6). Interventions that reduce central adiposity are therefore needed to prevent and treat metabolic disease and improve health span.

Aerobic exercise training can decrease central adiposity in individuals with overweight or obesity, regardless of age, sex, and ethnicity (8). However, the influence of aerobic exercise dose on central adiposity is equivocal, with some showing that greater doses do not decrease markers of central adiposity (9, 10) and others suggesting that reductions in VAT are improved at higher exercise doses (11, 12). Understanding the influence of exercise dose on central adiposity is crucial to help design optimal aerobic exercise regimens that enhance central adiposity outcomes. Thus, considering the conflicting findings, large randomized controlled trials are needed to test the effect of exercise dose on change in central adiposity markers, including VAT, in individuals with overweight and obesity.

Exercise-induced weight compensation, which is actual weight loss lower than weight loss predicted based on the energy expenditure of exercise, is common (13, 14). Weight compensation can occur because of multiple behavioral and physiological factors, although substantial weight compensation during exercise is primarily caused by elevations in energy intake (13). The relationship between compensation and central adiposity change during aerobic exercise is poorly understood. Some have demonstrated that reductions in VAT during high volumes of exercise are attenuated when compensation occurs through increased energy intake (15, 16), but the association between compensation and changes in central adiposity during exercise at doses similar to that recommended for health (700 to 1000 kcal/week) and weight loss and weight loss maintenance (∼2000 kcal/week) (17) has not been thoroughly studied. Moreover, the associations between central adiposity change and the mechanisms related to compensation—namely, increased energy intake, reduced energy expenditure and physical activity, and maladaptive eating attitudes and behaviors—during exercise training are not well understood. Assessing the compensation-related predictors of central adiposity change during exercise could pinpoint factors and/or constructs that may be targeted to enhance improvements in central adiposity.

This ancillary analysis had 2 primary aims. First, we tested the effect of aerobic exercise dose on changes in central adiposity in individuals with overweight or obesity. Second, we examined the associations between central adiposity changes and compensation during exercise training at guidelines akin to those recommended for health and weight loss and weight loss maintenance. As an exploratory aim, we assessed if mechanisms related to exercise-induced compensation predict VAT change during exercise.

## Methods

### Study Design

The methods of the Examination of Mechanisms of Exercise-Induced Weight Compensation (E-MECHANIC) study (ClinicalTrials.gov: NCT01264406) have been detailed elsewhere (13, 18). Briefly, the study was a 6-month randomized controlled trial that took place at Pennington Biomedical Research Center after institutional review board approval. After the provision of written informed consent, participants recruited to the study were randomized (N = 198) to 1 of 3 groups: a no-exercise control group, an exercise group that aimed to expend 8 kcal/kg/week (KKW) through exercise, or an exercise group that aimed to expend 20 KKW through exercise. The 8-KKW group reflected recommendations for general health (∼700 kcal/week), whereas the 20-KKW group reflected recommendations for weight loss and weight loss maintenance (∼1760 kcal/week) (13, 17). A biostatistician devised a 1:1:1 randomization ratio, and sex was stratified so that an equal number of males and females were randomized to each group. Randomization was concealed in an envelope until an interventionist or the study manager opened it with the participant. The participants and interventionists supervising exercise sessions were not blinded to group allocation, but the study investigators and the assessment team were because group allocation was not disclosed by the study manager or interventionists. Recruitment and data collection occurred from November 2010 to March 2015 (first participant enrolled 2011). Recruitment finished when the target sample size was recruited (13, 18).

### Participants

Sedentary (not exercising >20 minutes on ≥ 3 days/week) individuals living with overweight or obesity (body mass index [BMI], ≥ 25 kg/m^2^-≤ 45 kg/m^2^) who were otherwise healthy were recruited for the trial. Further details on the participant exclusion criteria have been reported (13).

### Intervention

Aerobic exercise training was conducted on a treadmill at an intensity that maintained participants within a heart rate range equivalent to 65% to 85% of baseline peak oxygen uptake. Participants in the 8-KKW group performed their complete exercise dose from the start. To acclimatize participants in the 20-KKW group, participants expended 8 KKW through exercise in week 1 and 14 KKW through exercise in week 2 before completing their complete dose (20 KKW of energy expenditure through exercise) from week 3 until the cessation of the study.

Exercise training was fully supervised and monitored. Participants were weighed weekly with a Tanita scale (Tanita Corporation, Arlington Heights, IL) and selected their exercise frequency (3, 4, or 5 sessions per week) to aid compliance. The energy expenditure target of each session was calculated by dividing the prescribed exercise dose (8 KKW or 20 KKW) by the exercise frequency. To meet the energy expenditure targets, the length of the exercise sessions varied. Real-time estimations of energy expenditure were calculated based on intensity and participant weight, and energy expenditure was measured periodically via a metabolic cart. Participants’ adherence to their exercise regimen was calculated as attained exercise energy expenditure divided by prescribed exercise energy expenditure.

The control group received health information (eg, stress management, benefits of healthy foods), although they were instructed to maintain their baseline physical activity.

### Outcome Measures

Body weight and waist circumference were measured at baseline and follow-up. Assessments of body composition were performed by dual-energy X-ray absorptiometry (DXA) at baseline and follow-up using Lunar iDXA with Encore software version 13.60 (GE Healthcare, Madison, WI, USA). DXA and Encore software quantified fat mass and body fat percentage for the whole body, trunk, arms, and legs, as well as VAT. The trunk-fat-to-limb-fat ratio (19) and VAT-to-total-fat ratio (20) were calculated as further assessments of central adiposity.

Compensation was calculated as actual weight change minus predicted weight change. Predicted weight at the end of the intervention was estimated using a validated dynamic energy balance model, which is a differential equation based on the first law of thermodynamics and accounts for metabolic adaptation and body composition changes during aerobic exercise training, overcoming the drawbacks of traditional predictions of weight during lifestyle regimens (21, 22). Predicted change in body weight and body composition are in response to the change in energy expenditure resulting from an increase in physical activity expenditure, as derived from the literature (22). Compensation was not included in the dynamic energy balance model. Indeed, the predicted body weight and body composition changes represent changes without compensation, and hence the difference between model body weight predictions and observed body weight reflects the degree of weight compensation.

Several measures were conducted at baseline and follow-up to assess mechanisms related to compensation (13). Energy intake was determined through doubly labeled water. In the primary outcome manuscript, change in energy intake with doubly labeled water was adjusted for resting metabolic rate, although estimates were also made without adjustments and with adjustments for body composition (13, 23). Results in the present analysis were similar with all estimates of energy intake; thus, change in energy intake with adjustment for resting metabolic rate is reported. Resting metabolic rate was examined via Max II metabolic carts (AEI Technologies), and steps per day were measured with SenseWear armbands (Body Media). The Eating Inventory assessed dietary restraint, disinhibition, and hunger (24). The Food Craving Inventory assessed intense desires to consume certain foods irrespective of hunger (only total score was used in the present analysis) (25). The Food Preference Questionnaire determined food preferences for certain food groups, as well as a fat preference score (only fat preference score was used in the current analysis) (26). Further, retrospective visual analogue scales assessed perceptions of appetite (27), the Compensatory Health Beliefs Scale measured compensatory health-related beliefs (eg, justifying eating because of exercise) (28), and the Activity Temperament Questionnaire examined participants’ tendency to move (29).

### Statistical Analysis

The current analysis assesses secondary endpoints of the E-MECHANIC trial. Because our secondary endpoints analysis requires follow-up data and adherence to the exercise intervention, participants assessed in the main analysis of the primary manuscript (ie, individuals with baseline and follow up data and ≥75% adherence to their exercise regimen) were considered (13). Including individuals with ≥75% adherence negated the influence of adherence as a possible confounder during between-group comparisons. In total, 171 participants satisfied the follow-up and adherence criteria, but 1 of the 171 participants did not have a baseline DXA measurement. As a result, our reference dataset for this study was restricted to 170 participants (Supplemental Figure 1 (30)).

All statistical analyses were performed in SPSS version 28, with the significance level set to α = .05. Differences in change scores among the 3 study groups were examined by 1-way analysis of covariance (ANCOVA), with adjustments for sex and age. Subgroup analyses were performed to examine variations in study group differences between: (1) those with high waist circumference (≥102 cm for males, ≥ 88 cm for females) (31) and healthy waist circumference (<102 cm for males, <88 cm for females) at baseline; (2) males and females; and (3) Black participants and participants of other races. These subgroup analyses were conducted via 2-way ANCOVA adjusted for sex (except for the male vs female subgroup analysis), age, and baseline values. Adjusted post hoc comparisons (Holm-Bonferroni) were performed when ANCOVA omnibus tests were significant to ascertain where differences lay. In exercisers, percent compensation (percentage compensation = [actual weight change – predicted weight change]/predicted weight loss) was calculated (13). Differences in waist circumference change, VAT change, VAT-to-total-fat ratio change, and trunk-fat-to-limb-fat ratio change were examined between those who showed positive compensation (percent compensation > 0%) and those with zero or negative compensation (percent compensation ≤ 0%) via 1-way ANCOVA adjusted for age, sex, and baseline values. Multiple linear regression models adjusted for sex, age, and baseline values also assessed the association between change in central adiposity indices and percent compensation. Pearson correlations tested the relationship between change in VAT and change in mechanisms related to compensation, and significant variables were then entered into a multiple linear regression model along with age, sex, and VAT at baseline to assess the predictors of VAT change in exercisers. We calculated absolute Cohen’s d effect size (ES) values to supplement between-group comparisons (32). Comparisons were considered negligible, small, medium, and large when ES values were <.20, .20 to .49, .50 to .79, and ≥.80, respectively, based on previous literature (32). Unless noted otherwise, values from inferential tests are estimated marginal mean (95% CI), whereas descriptive data are mean (SD).

## Results

### Descriptive

Descriptive characteristics of the participants included in the present analysis are shown in Table 1. Characteristics were similar when the full recruited sample (N = 198) was observed (data not shown). Most of the participants were female (N = 123; 72.4%) and White (N = 113; 66.5%). The mean age, weight, and BMI of the participants was 48.8 (±11.4) years, 88.6 (±15.4) kg, and 31.5 (±4.7) kg/m^2^, respectively.

**Table 1.** Descriptive characteristics of participants included in the per protocol analysis at baseline

		Control (N = 61)	8 KKW (N = 59)	20 KKW (N = 50)	All (N = 170)
Age, y		49.5 (10.8)	48.3 (11.1)	48.5 (12.5)	48.8 (11.4)
Sex					
	Male	16 (26.2)	16 (27.1)	15 (30.0)	47 (27.6)
	Female	45 (73.8)	43 (72.9)	35 (70.0)	123 (72.4)
Race					
	White	38 (62.3)	39 (66.1)	36 (72.0)	113 (66.5)
	Black	21 (34.4)	20 (33.9)	12 (24.0)	53 (31.2)
	Other	2 (3.3)	0 (.0)	2 (4.0)	4 (2.4)
Income					
	<$30 000	10 (16.4)	8 (13.6)	3 (6.0)	21 (12.4)
	$30 000-$49 999	9 (14.8)	7 (11.9)	7 (14.0)	23 (13.5)
	$50 000-$79 999	15 (24.6)	13 (22.0)	15 (30.0)	43 (25.3)
	$80 000-$99 999	12 (19.7)	10 (16.9)	9 (18.0)	31 (18.2)
	≥$100 000	14 (23.0)	20 (33.9)	15 (30.0)	49 (28.8)
	Don’t know or missing	1 (1.6)	1 (1.7)	1 (2.0)	3 (1.8)

Continuous data are mean (SD); categorical data are number (%).

Abbreviation: KKW, kcal/kg/week.

### Intervention Data

The 8-KKW group completed 101.0% (±6.3%) of prescribed exercise energy expenditure and the 20-KKW group completed 98.1% (±6.0%) of prescribed exercise energy expenditure, demonstrating the high adherence in both groups. Total energy expended by the 8-KKW group and 20-KKW group during exercise was 17 114 (±3175) kcal and 38 992 (±7308) kcal, respectively. On average, 680 (±123) kcal/week were expended by the 8-KKW group and 1521 (±263) kcal/week were expended by the 20-KKW group. Additional training data are shown in Supplemental Table 1 (30).

The average percent compensation shown by the 8-KKW group and the 20-KKW group was 70.0% (±129.2%) and 58.0% (±61.7%), respectively, and overall, 90 exercise participants (82.6%) showed positive compensation (ie, lost less weight than expected). Those who displayed positive compensation and those who displayed zero or negative compensation showed similar baseline characteristics (*P* ≥ .17; Supplemental Table 2 (30)).

### Weight and Total Body Composition Change

A difference between groups was identified for weight and BMI change (*P* = .02), with the 20-KKW group exhibiting a decrease compared with control (*P* < .03; ES ≥ .51; Table 2). Akin to results reported in the primary outcomes manuscript (13), total fat mass and body fat percent was reduced in the 20-KKW group compared with other groups (*P* < .05; ES ≥ .44), whereas no differences were seen for total lean body mass (*P* = .51; ES ≤ .22).

**Table 2.** Baseline values and 6-month change in weight, waist circumference, total adiposity, total lean mass, and regional adiposity in the control group, 8-KKW group, and 20-KKW group

		Control (N = 61)	8 KKW (N = 59)	20 KKW (N = 50)	*P*	Between-group ES		
						Control vs 8 KKW	Control vs 20 KKW	8 KKW vs 20 KKW
Weight, kg								
	Baseline	90.1 (86.2-94.0)	88.7 (84.8-92.7)	86.7 (82.4-91.0)				
	Change	−.3 (−1.0 to .4)	−.5 (−1.3 to .2)	−1.8 (−2.6 to −.9)*^b^*	.02*^a^*	.07	.51	.43
BMI, kg/m^2^								
	Baseline	32.3 (31.1-33.5)	31.4 (30.2-32.6)	30.6 (29.3-31.9)				
	Change	−.1 (−.4 to .2)	−.2 (−.5 to .1)	−.6 (−.9 to −.4)*^b^*	.02*^a^*	.10	.53	.43
Waist circumference, cm								
	Baseline	101.1 (98.0-104.3)	98.5 (95.3-101.7)	97.0 (93.6-100.5)				
	Change	.0 (−1.0 to 1.0)	−.7 (−1.7 to .4)	−1.3 (−2.4 to −.2)	.23	.15	.33	.17
Total fat mass, kg								
	Baseline	38.9 (36.3-41.4)	37.0 (34.5-39.6)	36.3 (33.5-39.0)				
	Change	.0 (−.6 to .7)	−.3 (−1.0 to .4)	−1.4 (−2.2 to −.7)*^c^*	.01*^a^*	.12	.57	.44
Total lean mass, kg								
	Baseline	48.2 (45.7-50.8)	48.8 (46.2-51.4)	47.4 (44.6-50.3)				
	Change	−.4 (−.7 to −.1)	−.2 (−.6 to .1)	−.1 (−.5 to .2)	.51	.13	.22	.09
Total body fat %								
	Baseline	43.2 (41.3-45.0)	41.6 (39.7-43.5)	41.8 (39.8-43.8)				
	Change	.1 (−.4 to .6)	−.1 (−.6 to .4)	−.9 (−1.5 to −.4)*^c^*	.02*^a^*	.09	.52	.44
VAT, kg								
	Baseline	1.40 (1.17-1.63)	1.25 (1.01-1.48)	1.30 (1.04-1.55)				
	Change	−.02 (−.07 to .04)	−.01 (−.07 to .04)	−.04 (−.10 to .02)	.71	.02	.13	.15
VAT-to-total-fat ratio								
	Baseline	.0361 (.0302-.0420)	.0336 (.0276-.0396)	.0363 (.0298-.0428)				
	Change	−.0008 (−.0016 to .0001)	−.0002 (−.0011 to .0007)	−.0004 (−.0014 to .0006)	.65	.17	.11	.06
Trunk fat mass, kg								
	Baseline	21.1 (19.6-22.7)	20.0 (18.4-21.6)	19.3 (17.5-21.0)				
	Change	.0 (−.5 to .4)	−.3 (−.8 to .2)	−.8 (−1.3 to −.3)	.07	.14	.43	.29
Trunk fat %								
	Baseline	46.9 (45.0-48.7)	45.0 (43.2-46.9)	44.8 (42.7-46.8)				
	Change	−.1 (−.7 to .5)	−.2 (−.8 to .4)	−1.1 (−1.8 to −.4)	.06	.04	.42	.38
Arms fat mass, kg								
	Baseline	4.01 (3.72-4.30)	3.84 (3.54-4.14)	3.60 (3.28-3.93)				
	Change	.06 (−.02 to .15)	.06 (−.03 to .15)	−.07 (−.17 to .02)	.06	.01	.41	.39
Arms fat %								
	Baseline	39.9 (37.5-42.3)	38.5 (36.1-40.9)	38.4 (35.7-41.0)				
	Change	.7 (.2 to 1.1)	.7 (.2 to 1.1)	−.1 (−.5 to .4)	.04*^a^*	.01	.42	.43
Legs fat mass, kg								
	Baseline	12.74 (11.70-13.77)	12.19 (11.14-13.24)	12.42 (11.28-13.57)				
	Change	−.01 (−.28 to .26)	−.05 (−.33 to .22)	−.54 (−.83 to −.24)*^c^*	.02*^a^*	.04	.50	.45
Legs fat %								
	Baseline	42.0 (39.6-44.3)	41.0 (38.6-43.3)	41.6 (39.0-44.1)				
	Change	.3 (−.2 to .7)	−.2 (−.7 to .2)	−1.0 (−1.5 to −.5)*^c^*	<.01*^a^*	.26	.72	.46
Trunk-fat-to-limb-fat ratio								
	Baseline	1.32 (1.22-1.42)	1.28 (1.18-1.38)	1.27 (1.16-1.38)				
	Change	−.03 (−.06 to .00)	−.02 (−.05 to .01)	−.02 (−.05 to .02)	.90	.06	.08	.03

Data are estimated marginal mean (95% CI). Change values are adjusted for age and sex.

Abbreviations: ANCOVA, analysis of covariance; BMI, body mass index; ES, effect size; KKW, kcal/kg/week; VAT, visceral adipose tissue.

^*a*^Significant ANCOVA omnibus test for study group (*P* < .05).

^*b*^Significantly different from control group (*P* < .05).

^*c*^Significantly different from the control group and 8-KKW group (*P* < .05).

### Regional Adiposity Change

No significant between-group difference was observed for change in waist circumference (*P* = .23), despite confidence interval data indicating a within-group reduction in the 20-KKW group (Table 2). Changes in VAT, the VAT-to-total-fat ratio, and the trunk-fat-to-limb-fat ratio were similar in all groups, with negligible ES values seen (all *P* ≥ .65; all ES ≤ .17). In spite of CI data showing reductions in trunk fat mass and trunk percent fat in the 20-KKW group, between-group differences were not significantly different and small to negligible ES values were revealed (all *P* ≥ .06; all ES ≤ .43). Arm percent fat, leg fat mass, and leg percent fat were statistically different between groups (all *P* ≤ .04). Post hoc tests for leg fat mass and leg percent fat indicated a greater reduction in leg fat in the 20-KKW group compared with other groups (*P* ≤ .04), although no significant between-group variations were observed for arm percent fat following adjustment (*P* > .08: Table 2). Results from subgroup analyses are shown in Supplemental Tables 3-5 (30). The effect of study group on changes in weight, BMI, waist circumference, and DXA endpoints was not modified by waist circumference at baseline or race (all *P* for interaction ≥ .07). There was a 2-way interaction between study group and sex for VAT-to-total-fat ratio change and trunk-fat-to-limb-fat ratio change (*P* for interaction < .05), but following adjustments for multiple comparisons, no significant between-group differences were seen when males and females were analyzed separately (*P* > .05).

**Figure 1.**
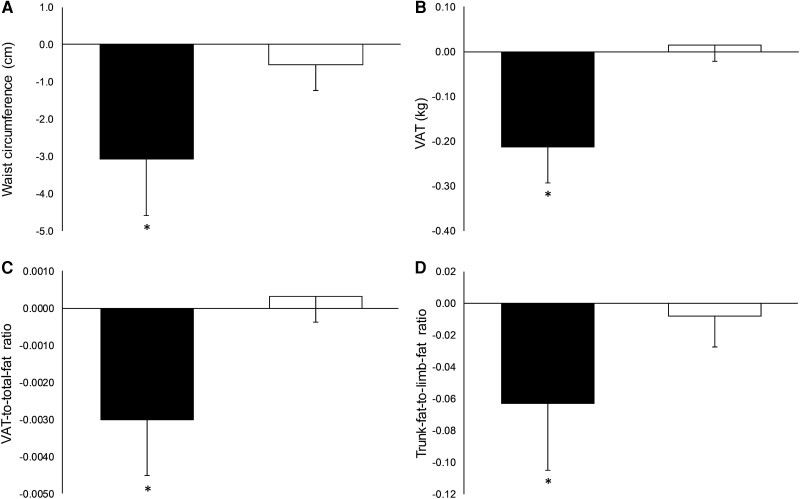
Change in waist circumference (A), VAT (B), VAT-to-total-fat ratio (C), and trunk-fat-to-limb-fat ratio (D) in individuals who displayed zero or negative weight compensation (ie, those who lost more or equal weight to that which was predicted) (N = 19) and individuals who displayed positive weight compensation (ie, those who lost less weight than predicted) (N = 90) during exercise. Abbreviations: ANCOVA, analysis of covariance; VAT, visceral adipose tissue. Black bars are individuals who showed zero or negative compensation; white bars are individuals who showed positive compensation. Data are estimated marginal means (95% CI) adjusted for age, sex, and baseline values. *Significant ANCOVA omnibus comparison between those who displayed zero or negative compensation and those who displayed positive compensation (*P* < .05).

Individuals with positive weight compensation displayed a 2.5-cm (95% CI, .8-4.2; ES = .76), .23-kg (95% CI, .14-.31; ES = 1.31), .0033 (95% CI, .0017-.0050; ES = 1.02), and .06 (95% CI, .01-.10; ES = .60) increase in waist circumference, VAT, the VAT-to-total-fat ratio, and the trunk-fat-to-limb-fat ratio, respectively, compared with individuals with zero or negative weight compensation (all *P* ≤ .02; Fig. 1). Multiple linear regression analyses showed greater weight compensation during exercise training was associated with increases in waist circumference, VAT, the VAT-to-total-fat ratio, and the trunk-fat-to-limb-fat ratio (all β ≥ .24; *P* ≤ .01; Supplemental Table 6 (30)).

In Pearson correlation analyses, change in compensatory health beliefs (*r* = .20; *P* = .04) and retrospective desire to eat (*r* = .23; *P* = .02) were related with VAT change during exercise (Supplemental Table 7 (30)); hence, these variables were entered into the multiple linear regression analysis. This regression analysis revealed that retrospective desire to eat was a positive predictor of VAT change (β = .21; *P* = .03; Table 3). This model also suggested that compensatory health beliefs was not a significant predictor of VAT change, although a similar standardized β was observed (β = .16; *P* = .09).

**Table 3.** Multiple linear regression analysis for association between retrospective desire to eat and compensatory health beliefs, and change in VAT

		*R* ^2^	B	95% CI	β	*P*
VAT (kg)		.115				
	Retrospective VAS, desire to eat		.0023	(.0002 to .0045)	.21	.03*^a^*
	Compensatory health beliefs		.0047	(−.0007 to .0101)	.16	.09
	Age		.0011	(−.0023 to .0046)	.07	.51
	Sex*^b^*		−.0868	(−.1939 to .0202)	−.20	.11
	VAT at baseline		−.0001	(−.0001 to .0000)	−.23	.07

Abbreviations: VAS, visual analogue scale; VAT, visceral adipose tissue.

^*a*^Statistically significant (*P* < .05).

^*b*^Male = 1, female = 2.

## Discussion

Overall, in this ancillary analysis of a large, 6-month randomized controlled trial in individuals with overweight and obesity, we showed negligible differences in central adiposity change between a no-exercise control group and 2 aerobic exercise groups—1 similar to guidelines recommended for health and 1 similar to guidelines recommended for weight loss and weight loss maintenance. We also showed that exercisers who displayed positive weight compensation (ie, lost less weight than predicted) showed reduced improvements in central adiposity relative to those who did not compensate. These results indicate that exercise dose has no significant impact on central adiposity, and that significant compensation is likely to negate central adiposity improvements during exercise at guidelines for health and weight loss and weight loss maintenance.

Greater energy expenditure during aerobic exercise training leads to increased weight loss because of a higher energy deficit (33), but the influence of aerobic exercise dose on central adiposity is equivocal over 6 months or more. Although Recchia and colleagues demonstrated that greater energy expenditure through aerobic exercise leads to small yet significant improvements in central adiposity (12), a smaller trial showed that increasing exercise dose does not lead to improvements in VAT (9), and others have demonstrated no differences in waist circumference between individuals performing exercise at 50%, 100%, and 150% of guidelines (10). Results from our trial displayed no significant differences in central adiposity changes over 6 months between a control group and 2 groups exercising at doses resembling that recommended for health (680 kcal/week) and for weight loss and weight loss maintenance (1521 kcal/week). We did observe significant reductions in total fat and adiposity in other noncentral regions in the 20-KKW group, which could provide metabolic benefits for individuals with overweight and obesity (34). Confidence intervals also indicated that the 20-KKW group demonstrated a reduction in some central adiposity indices (eg, waist circumference, trunk fat), and between-group significance levels for trunk fat mass and trunk percent fat were close to the significance threshold. However, estimated marginal mean and effect size data show variations in central adiposity between groups are negligible or small at best. The 20-KKW group, for example, exhibited a 1.3-cm and .7-cm decrease in waist circumference compared with the control group and the 8-KKW group, respectively, and these differences are considered clinically unimportant (35, 36) based on associations between waist circumference change and metabolic disease (37) and mortality (38). Thus, on balance, though relatively small levels of physical activity can improve central adiposity (10, 39), we believe exercise doses that expended ∼700 kcal/week and ∼1500 kcal/week induced clinically trivial changes in central adiposity during this trial. Doses with even greater differences in exercise-induced energy expenditure may be required to detect clinically meaningful improvements in central adiposity.

A reason why exercise groups exhibited negligible changes in central adiposity compared with control in our study could be the compensation displayed by exercise groups. The majority (82.6%) of exercisers exhibited positive weight compensation (ie, lost less weight than expected) and these participants displayed a 2.5-cm and .23-kg increase in waist circumference and VAT, respectively, compared with those who did not show positive weight compensation. Although few have examined the link between compensation and changes in central adiposity, 2 studies in individuals with high waist circumference showed that aerobic exercise without weight loss (ie, with compensation) led to attenuated reductions in central adiposity relative to exercise with weight loss (ie, without compensation) in males (15) and females (16), supporting our findings. Nonetheless, contrary to our results, these studies still found significant central adiposity improvements in exercisers who compensated (15, 16). That these earlier studies solely recruited individuals with high waist circumference is unlikely to explain why the previous studies saw improvements in central adiposity in individuals who compensated and we did not, as we found no exercise-induced differences in central adiposity change between those with high and healthy waist circumference at baseline. Rather, the discrepancies could occur because the previous studies were only 3 months and/or they implemented far greater exercise energy expenditures of 3500 kcal/week in women (16) and 4900 kcal/week in men (15). These findings could collectively indicate that exercise in the presence of significant weight compensation improves central adiposity during short interventions in which exercise volumes are high, but not during medium to long-term regimens in which exercise performed is similar to that recommended for health and weight loss and weight loss maintenance.

By highlighting the compensatory mechanisms that predict central adiposity change during exercise, effective strategies can be developed to improve central adiposity outcomes during exercise. Several mechanisms could drive positive weight compensation during exercise training: an increase in energy intake, maladaptive changes in eating behaviors and physical activity patterns, and reductions in exercise and nonexercise energy expenditure (13). In this analysis, an increase in desire to eat positively predicted change in VAT. A similar association between change in VAT and compensatory health beliefs (eg, justifying an eating episode because of exercise) was also observed, albeit the coefficient was smaller and tended to be significant. Along with findings from the primary outcome manuscript that showed exercise-induced elevations in energy intake (13), these results may indicate that changes in eating habits attenuated reductions in central adiposity during exercise. This is in line with previous studies (15, 16) and implies that strategies targeting desire to eat and compensatory behaviors during exercise could enhance central adiposity outcomes. Akin to other regimens (40), such strategies could include behavioral sessions that help manage appetite by encouraging participants to increase consumption of foods with low energy density (41). Additional strategies and sessions focussing on meal planning and portion and stimulus control could also decrease compensatory meals and/or snacking during exercise training (40). Nevertheless, it should be acknowledged that more work is needed to elucidate the role of compensatory behaviors in modifying central adiposity changes during exercise because most compensatory behaviors (including energy intake) were not related to VAT and our models explained a small proportion of VAT variance.

A strength of the present analysis is that it comprises energy intake, energy expenditure, and physical activity outcomes assessed with gold-standard techniques, as well as questionnaires examining eating attitudes and behaviors. One limitation is that we did not use computed tomography or magnetic resonance imaging, which are considered gold standard tools for VAT assessment. Although VAT assessments via DXA are linked to computed tomography-derived measurements (42) and our primary findings were consistent among several indices related to VAT, future studies using computed tomography or magnetic resonance imaging are warranted. Additionally, because we did not assess potential physiological mediators, further studies are needed to examine the mechanisms underpinning our findings. Another limitation is that most of the sample were female and white, so it is possible we were underpowered to detect consistent and significant interactions in our subgroup analyses. It is also noteworthy that this manuscript reports an ancillary project, though it should be acknowledged that it used data from a large, randomized control trial where exercise adherence was excellent and exercise dose was fastidiously supervised and monitored.

Taken together, the present study indicates that in the presence of significant weight compensation, there are negligible differences in central adiposity change during aerobic exercise at doses similar to that recommended for health and weight loss and weight loss maintenance in individuals with overweight or obesity. Moreover, higher weight compensation was associated with reduced improvements in central adiposity, and exercisers with increased subjective desire to eat exhibited poorer change in central adiposity. During exercise at guidelines for health and weight loss and weight loss maintenance, exercise-induced compensation should be treated and reduced in individuals with overweight or obesity to enhance central adiposity reductions, potentially through strategies that manage appetite and compensatory food behaviors.

## Data Availability

Some or all datasets generated during and/or analyzed during the current study are not publicly available but are available from the corresponding author on reasonable request. **Clinical trials registration:** NCT01264406.

## Abbreviations

ANCOVA: analysis of covariance
BMI: body mass index
DXA: dual-energy X-ray absorptiometry
E-MECHANIC: Examination of Mechanisms of Exercise-Induced Weight Compensation
ES: effect size
KKW: kcal/kg/week
VAT: visceral adipose tissue
